# Polarity-dependent modulation of sleep oscillations and cortical excitability in aging

**DOI:** 10.3389/fnagi.2025.1704130

**Published:** 2026-01-15

**Authors:** Buse Dikici, Robert Malinowski, Jan-Bernhard Kordaß, Klaus Obermayer, Julia Ladenbauer, Agnes Flöel

**Affiliations:** 1 Department of Neurology, Universitätsmedizin Greifswald, Greifswald, Germany; 2 Technische Universität Berlin, Berlin, Germany; 3 Bernstein Center for Computational Neuroscience Berlin, Berlin, Germany

**Keywords:** excitation/inhibition (E/I) balance, memory consolidation, phase amplitude coupling, sleep, sleep spindle, slow oscillation, slow oscillatory tDCS

## Abstract

**Background:**

During non-rapid eye movement (NREM) sleep, cortical slow oscillation (SO; <1 Hz) and thalamic sleep spindle activity (12–15 Hz) interact through precise phase coupling to support memory consolidation. Slow oscillatory transcranial direct current stimulation (so-tDCS) can modulate these oscillations. Traditionally, anodal so-tDCS is used to depolarize the cortex during SO up-states, thereby promoting SO activity and SO-spindle coupling. However, intracranial findings suggest that SO down-states, characterized by cortical hyperpolarization, can trigger thalamic spindle bursts. This raises the hypothesis that cathodal so-tDCS, by promoting hyperpolarization, could selectively enhance down-states and more effectively improve SO-spindle coupling.

**Methods:**

We tested this hypothesis in 22 healthy older adults, a population known to exhibit diminished NREM oscillatory activity. Each participant received cathodal, anodal, and sham so-tDCS in separate nap sleep sessions. We quantified SO and spindle characteristics, their temporal coupling, and cortical excitation/inhibition (E/I) balance using EEG spectral slope. We also assessed individual circadian preference (chronotype) as a potential moderator.

**Results:**

We found that anodal so-tDCS improved SO-spindle synchrony and increased spindle power over sham in participants with intermediate or evening chronotypes, while cathodal so-tDCS did not enhance these oscillatory measures compared to sham, despite prolonging SO down-states. Anodal so-tDCS also shifted E/I balance toward increased excitability, indicating increased cortical excitability, whereas cathodal so-tDCS did not produce the anticipated opposite shift.

**Conclusion:**

In summary, anodal, but not cathodal, so-tDCS effectively enhanced thalamocortical interactions underlying memory consolidation. Furthermore, these findings highlight the importance of individual factors such as chronotype in brain stimulation responsiveness.

## Introduction

Cortical slow oscillations (SOs; <1 Hz) and thalamocortical sleep spindles (12–15 Hz) are core features of non-rapid eye movement (NREM) sleep. These rhythms are not only temporally structured but also functionally interdependent: SOs temporally coordinate the emergence of spindles, with spindle bursts preferentially occurring near the SO up-state, when cortical excitability is high (Klinzing et al., 2019). This precise phase coupling is thought to underlie systems-level memory consolidation and was shown to be diminished in older adults (Helfrich et al., 2018; Ladenbauer et al., 2021).

The ability to modulate these oscillatory dynamics noninvasively has opened new avenues for probing and potentially enhancing sleep-related memory consolidation. A promising non-invasive approach is slow oscillatory transcranial direct current stimulation (so-tDCS) with anodal polarity (Fehér et al., 2021; Ladenbauer et al., 2017). Delivered bifrontally during NREM sleep, the sinusoidal current with SO frequency (<1 Hz) is thought to depolarize cortical neurons toward firing threshold (Nitsche and Paulus, 2000), thereby facilitating SO up-states and entraining the brain’s endogenous SO rhythm (Marshall et al., 2006). Consistent with this mechanism, anodal so-tDCS has been demonstrated to enhance SO activity while also promoting spindle activity (Marshall et al., 2006; Westerberg et al., 2015; Prehn-Kristensen et al., 2014; Ladenbauer et al., 2016) and improving their precisely timed phase coupling (Ladenbauer et al., 2023). Concurrently, anodal so-tDCS has been shown to improve memory retention performance, with SO-spindle coupling being most informative for this improvement (Ladenbauer et al., 2017).

The rationale for using anodal polarity was originally grounded in observations of surface negative direct current (DC) potential shifts during transitions into slow wave sleep (Marshall et al., 1998). These shifts suggest increased cortical excitability and showed strong associations with spindle and slow wave activity during NREM sleep (Marshall et al., 2003). As a result, anodal so-tDCS has become the standard tDCS approach for enhancing NREM sleep oscillations during sleep (Westerberg et al., 2015; Prehn-Kristensen et al., 2014; Paßmann et al., 2016).

However, recent findings from intracranial recordings in humans challenge the choice of anodal polarity for so-tDCS application. Mak-McCully et al. (2017) identified cortical hyperpolarized down-states during SO events as key triggers for thalamic down-states, which in turn initiate spindle bursts that feed back to the cortex, timing cortical spindle activity to the late rising phase of the SO. These results suggest that cortical hyperpolarizations, which produce thalamic down-states, strongly affect the occurrence and timing of thalamo-cortical spindles. From this mechanistic perspective, biasing cortical networks toward hyperpolarization during the down-state via cathodal so-tDCS could theoretically engage this cascade more directly than promoting cortical depolarization.

Moreover, stimulation approaches that enhance cortical hyperpolarization may be especially beneficial in aging populations. Neuronal “hyperactivity” (i.e., more cortical depolarization) is commonly observed in older compared to younger adults (Voytek et al., 2015), and is especially pronounced in pathological aging, such as Alzheimer’s disease (Busche and Konnerth, 2016). This hyperactivity reflects a disruption in cortical excitation/inhibition (E/I) balance, a process associated with cognitive decline (Li et al., 2025). By promoting hyperpolarizing phases during NREM sleep, cathodal so-tDCS may further help restore E/I balance and potentially reduce age-related cognitive impairments (Krause et al., 2013). Furthermore, since the efficacy of anodal so-tDCS during sleep can show considerable variability across individuals (Ladenbauer et al., 2017), potentially due to intrinsic differences in participants’ brain states or physiology, we aimed to address this issue in the given study. One emerging factor is chronotype, a person’s circadian preference for morning or evening activity. Salehinejad et al. (2021) found that morning-type and evening-type individuals exhibit differences in baseline cortical physiology and in their response to tDCS-induced plasticity during wakefulness at different times of day. Specifically, they observed that cortical E/I balance was shifted toward excitation at each chronotype’s preferred time, and both anodal as well as cathodal tDCS effects were stronger at the preferred time (with opposite effects). While sleep stages strongly reshape E/I balance, the circadian processes underlying chronotype likely continue to modulate neuronal excitability throughout sleep, particularly during daytime naps. Therefore, chronotype-related differences present during wakefulness may likely carry into nap sleep and may thus modulate the efficacy of polarity-specific so-tDCS effects during sleep. However, this possibility remains unexplored.

In the present study, we investigated the effects of cathodal so-tDCS on NREM sleep oscillations in a within-subject experiment in healthy older adults. Our goals were three-fold: first, to determine the influence of cathodal so-tDCS during a 90-min daytime nap on (i) SO down-states, (ii) spindle activity, (iii) precision of slow oscillation-spindle coupling, and (iv) cortical E/I balance indexed by EEG spectral slope as an indication for changes in cortical excitability (Gao et al., 2017); second, to compare effects of cathodal and anodal so-tDCS (both relative to sham) on sleep microstructure; and third, given recent evidence that individual circadian preference (chronotype) can influence neuroplastic responses to continuous tDCS during wakefulness (Salehinejad et al., 2021; Salehinejad et al., 2022), to assess whether individual chronotype (as measured by MEQ values) moderates the effects of so-tDCS of either polarity during sleep.

## Materials and methods

### Participants

**Figure 1:**
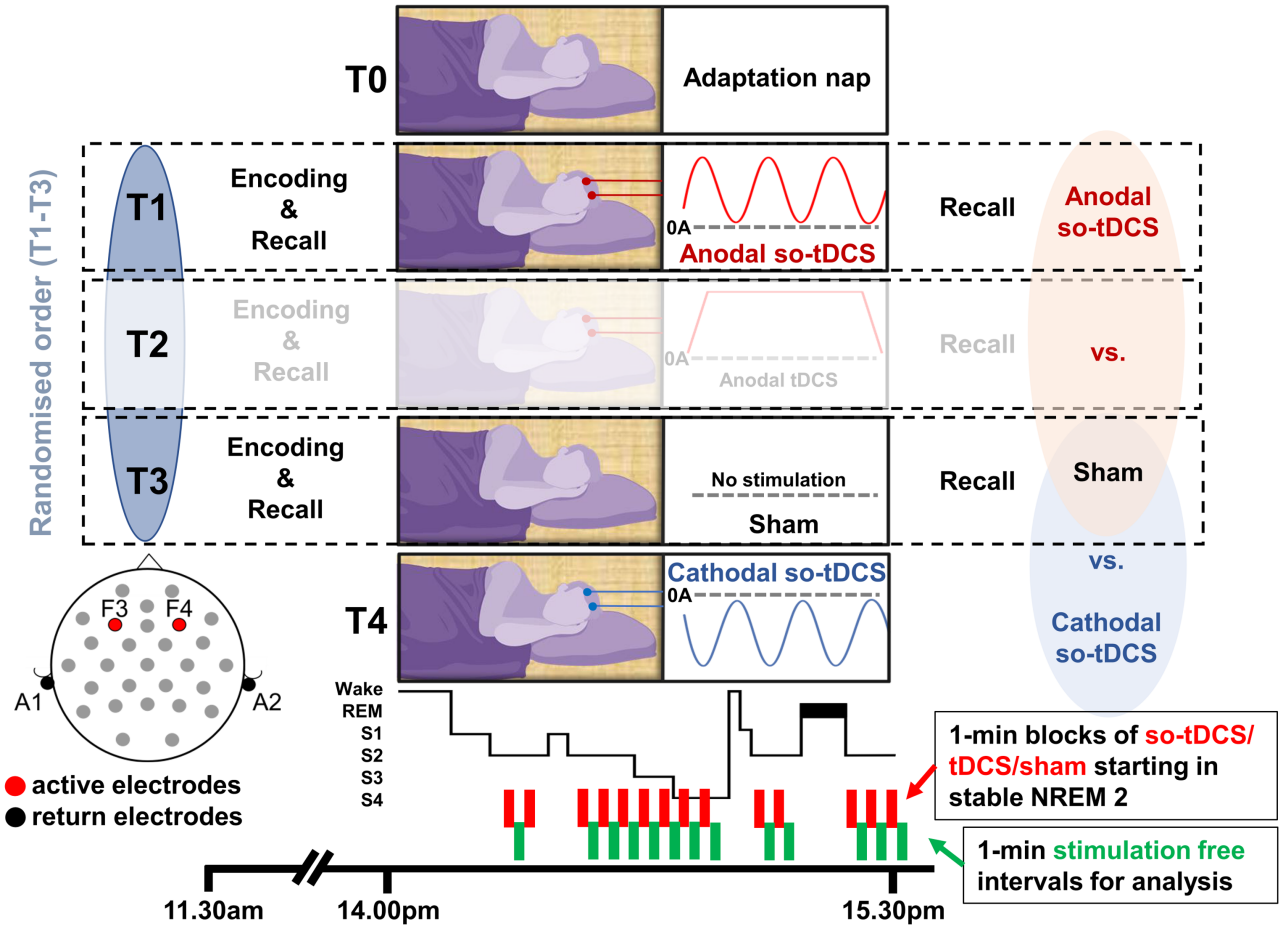
Experimental procedure and stimulation protocol. Following an adaptation nap, participants completed four experimental sessions separated by ≥1 week: three randomized conditions (anodal so-tDCS, constant anodal tDCS, sham) plus a non-randomized cathodal so-tDCS session (always last). This manuscript analyzes three conditions examining polarity-specific effects: anodal so-tDCS, cathodal so-tDCS, and sham. Oscillatory tDCS (0.75 Hz, 5–255 μA) was delivered bilaterally via frontal electrodes (F3, F4; mastoid returns) in 1-min blocks during N2/N3 sleep, with ≥ 1.5-min inter-block intervals. Sessions included a 90-min nap opportunity with EEG recording (2:00 p.m.), flanked by memory testing in the anodal, constant anodal, and sham conditions but not in the cathodal condition.

In total, 28 healthy older adults (age 55–79 years, mean 65.9 ± SD 6.8 years) participated in the cathodal so-tDCS condition of a larger study examining four tES protocols (see Figure 1 for experimental procedure). The sample included habitual nappers (*n* = 13) and non-nappers (*n* = 9) who showed comparable nap sleep duration (*p* = 0.507) and efficiency (*p* = 0.978). Inclusion required sufficient sleep during previous nap sessions (with anodal so-tDCS and sham), defined as completing at least seven so-tDCS /sham blocks during the nap. Six participants were excluded due to insufficient sleep during the cathodal so-tDCS session, leaving 22 participants for final analysis. Participants were screened for eligibility via a structured telephone interview to ensure that they did not have a history of severe, untreated neurological, psychiatric, or sleep disorders. On site, exclusion criteria additionally encompassed intake of medications acting on the central nervous system (e.g., antipsychotics, antidepressants, antihistamines, benzodiazepines, or any sleep-inducing medications), severe hearing or vision impairment, alcohol or substance abuse, a contraindication or inability to undergo MR imaging, and brain pathologies identified on MRI scan (brain tumor and stroke), as well as a lack of German language skills. Furthermore, participants underwent extensive baseline assessments (see Supplemental Information for more details and Supplementary Table S1 for baseline characteristics). The study was ethically approved, adhered to the Declaration of Helsinki, and all participants provided written informed consent and received compensation.

### Procedure

Nap sessions took place at the sleep laboratory of the Department of Neurology, University Medicine Greifswald, Germany. The complete experimental protocol comprised four sessions after an adaptation nap: three conditions (anodal so-tDCS, constant anodal tDCS, and sham) administered in randomized order, plus a fourth non-randomized session with cathodal so-tDCS, which was always conducted last. This fourth session was added after data collection had commenced to address an additional research question regarding polarity-specific effects on sleep oscillations. The current manuscript focuses specifically on these polarity-specific effects and therefore analyzes three conditions: anodal so-tDCS, cathodal so-tDCS, and sham. The constant anodal tDCS condition (vs. anodal oscillatory tDCS), which addresses questions about entrainment versus plasticity mechanisms, will be reported separately. Each session was separated by at least 1 week.

Upon arrival at the sleep laboratory at 11:30 a.m., participants were prepared for EEG recordings and then completed the learning phase of two computerized declarative memory tasks consisting of an object-location task and a verbal paired-associate learning task. At 2:00 p.m., participants were asked to attempt to sleep for a period of 90 min, and subsequently, participants performed the retrieval phase of the memory tasks. The last cathodal so-tDCS session was conducted in an identical manner, with the exception that the experimental session began at 12:30 p.m., as no memory tasks were tested for this condition.

### Brain stimulation

A setup of two battery-driven stimulators (DC-Stimulator; NeuroConn, Ilmenau, Germany) was used to deliver the oscillatory current stimulation. Stimulation electrodes (8 mm diameter) were placed bilaterally at frontal locations (F3, F4), with return electrodes on ipsilateral mastoids (8 mm diameter; M1, M2). The current, of either anodal or cathodal polarity, followed a sinusoidal waveform oscillating between 5 and 255 μA, generating a maximum current density of 0.507 mA/cm^2^ per hemisphere. Stimulation began after participants exhibited stable NREM stage 2 (N2) sleep for at least 4 min (stable N2 defined as no transitions back to N1 or wake lasting longer than one epoch/30 s). Stimulation was delivered in repeated 1-min blocks (7–15 blocks, min. 7 were required for inclusion), each separated by at least 1.5 min. Sleep stages were monitored after each stimulation block through online scoring, and each stimulation block was only initiated when participants were in N2 or N3 sleep. Intermediate stimulation-free intervals were prolonged if participants transitioned out of N2/N3, and resumed only after a stable return to N2 for at least 1 min (no transition). This intermittent block design follows established sleep-stimulation protocols (Marshall et al., 2006) and aligns with recent evidence that even brief so-tDCS epochs effectively modulate slow-oscillation dynamics (Ladenbauer et al., 2023). Shorter trains are also well suited to the more variable NREM architecture of nap sleep, enabling stimulation to be delivered in a state-dependent manner while avoiding transitional or wake periods. Short stimulation blocks also provide methodological advantages, as the stimulation-free intervals offer artifact-free periods for quantifying sleep physiology and increase the number of analyzable epochs. Sham sessions followed identical temporal patterns without actual stimulation. After all naps, participants were asked whether they had perceived any sensations during the naps.

### Sleep monitoring and EEG preprocessing

The EEG cap (actiCAP, Brain Products GmbH, Gilching, Germany; 64-channels) for sleep monitoring was prepared according to the 10–20 international EEG system after tracking the electrode positions on the cap with a neuronavigation system (Rogue Research, Montréal, Canada) to ensure identical cap and stimulation electrode placement in each nap session. During recording, EEG data were referenced to the nose electrode, with EOG and chin EMG recorded for sleep staging (500 Hz sampling rate). After recording, the YASA sleep scoring algorithm (Vallat and Walker, 2021) was used to conduct offline sleep staging on 30-s epochs. All epochs with high uncertainty (confidence threshold <55%) were manually inspected and scored by an expert scorer. Additionally, the entire sleep EEG recording was reviewed for accuracy, with manual corrections applied where necessary. As AASM recommended, EEG derivations were partly occupied by stimulation electrodes, we used the following channels for offline sleep staging: F1, C3, O1, or F2, C4, O2 in case of noisy channels; in addition to EOG and MEG. Stimulation epochs were not scored due to strong artifacts in the EEG signal, a method also applied to the corresponding epochs in the sham session. Preprocessing and analysis of EEG data were conducted with MNE-python (v1.7.1) (Gramfort et al., 2013) using custom scripts based on the open-source code by Hanna (2023),^1^ which we adapted for our needs (Kordaß and Malinowski, 2025).^2^ After applying a notch filter at 50 Hz and its harmonics, data were downsampled to 200 Hz, followed by automated annotation of so-tDCS/sham intervals, bad-channel identification (ANOAR package) (Hanna and Pulvermüller, 2018), and visual artifact rejection.

### EEG analyses

Analyses focused primarily on 1-min intervals following each so-tDCS or sham block at fronto-central derivations (Fz, or FC1 or FC2 in case of noise).

#### Spectral power analyses

YASA (Vallat and Walker, 2021) was used to perform spectral power analysis in the SO (0.5–1 Hz) and spindle frequency range (12–15 Hz). Power spectral density (PSD) was computed using Welch’s method on consecutive 4-s epochs with 50% overlap and a Hamming window, resulting in a frequency bin resolution of 0.25 Hz. The absolute band power for SO (0.5–1 Hz) and spindle (12–15 Hz) frequency ranges was calculated by integrating the power spectrum within these frequency bands (area under the curve). To obtain relative power, the absolute band powers were then normalized by dividing by the total power within the 0.5–35 Hz frequency range.

#### SO event detection

SO detection followed practices established in the literature (Ladenbauer et al., 2017; Ladenbauer et al., 2023; Mölle et al., 2009). Frequencies up to ~1.25 Hz were included to capture SO cycles with durations as brief as ~0.8 s, in line with standard SO event definitions based on zero-crossing intervals (Staresina et al., 2015; Mölle et al., 2009). In brief, the signal was filtered using a finite impulse response (FIR) bandpass filter with a frequency range of 0.16–1.25 Hz, after which all negative-to-positive zero-crossings in the signal were identified. The time between two successive negative zero-crossings was measured, as well as the amplitude difference between the most negative point (down-state trough) after the first positive-to-negative zero-crossing, and the most positive point (up-state peak) after the next negative-to-positive crossing. SO events were marked if this trough-peak amplitude exceeded each participant’s 65th percentile of all detected zero-crossings, and the duration between two consecutive positive-to-negative zero-crossings was between 0.8 and 2 s. In accordance with Muehlroth and Werkle-Bergner (2020), who stress that age-related reductions in slow-wave amplitude necessitate age-adapted detection approaches, we used an individualized percentile-based threshold to avoid systematic under-detection associated with fixed absolute thresholds (e.g., 75 μV) in older adults. This individualized amplitude thresholding procedure follows the implementation described in Ladenbauer et al. (2023), ensuring that physiologically meaningful lower-amplitude slow oscillation characteristics of aging are reliably captured.

#### Time-frequency representation analysis

Time-frequency representations (TFRs) were calculated for each SO event epoch (5 s epoch) using the Morlet wavelet transform with 5 cycles, after data were first down-sampled to 50 Hz. It was applied for frequencies of 10–20 Hz in steps of 0.2 Hz to provide robust power estimates across the spindle frequency range and surrounding frequencies (Ngo et al., 2020). The first and last 150 ms of each epoch were cut off to remove edge effects. After calculating TFR within the spindle band for each SO epoch, baseline adjustment followed the z-score method, such that the mean and standard deviation of the time period −2.35 to −1.5 s were used in a z-score transformation of the entire SO epoch. First, a two-tailed paired-samples *t*-test was conducted to determine statistical significance between the conditions at the group level. Next, a cluster-based permutation test (1,024 iterations) was applied using the threshold-free cluster enhancement (TFCE) method to account for multiple comparisons and to detect significant clusters. The significance threshold was set at 0.05.

#### SO-spindle coupling

In addition to TFR analyses, slow oscillation-spindle coupling was quantified through event-locked phase-amplitude coupling analyses. First, the normalized SO event segments were filtered in the SO band (0.5–1.25 Hz), and the instantaneous phase angle was extracted using a wavelet transformation. Then, the same segments were filtered in the spindle band (12–15 Hz), and the instantaneous amplitude was also extracted with a wavelet transformation. To avoid filter edge artifacts, we only considered the time range −2 to 2 s, centered on the SO trough. We next extracted the maximal spindle amplitude and corresponding SO phase angle for every subject and SO event. This yielded a distribution of spindle-at-peak phase angles for each participant and condition (e.g., 0° = positive peak, 90° = midpoint of the falling phase, 180° = negative trough, 270° = midpoint of the rising phase). From these distributions, we computed two measures: (1) the mean coupling phase angle (circular measure) and (2) the coupling strength, quantified as the resultant vector length. Mean coupling phase angles were compared between conditions using circular statistics (Watson–Williams tests). Signal decomposition was carried out with the Python package Tensorpac 0.6.5 27.

#### Cortical excitation/inhibition (E/I) balance

To test whether cathodal vs. anodal so-tDCS stimulation induced divergent shifts in cortical excitability during post-stimulation intervals, we extracted the spectral slope x (the negative exponent of the 1/fx decay function) from the 1 to 45 Hz frequency range of PSD using the FOOOF package (Donoghue et al., 2020) with the ‘fixed’ aperiodic mode (similar to Lendner et al., 2023). The spectral slope of the log–log PSD was estimated for each interval and averaged per condition within each subject. A more negative spectral slope (i.e., a steeper decline of power at higher frequencies) indicates reduced high-frequency activity relative to low-frequency – often interpreted as a shift toward greater inhibition relative to excitation, whereas a flatter slope implies more high-frequency power and potentially greater excitatory tone. Additionally, checked 1-min pre-stimulation baseline segments to confirm no inherent differences before stimulation. Regression analyses between spectral slope, spindle activity, and coupling measures were also explored.

#### Individual morning–evening preference

Furthermore, we examined whether individual chronotype moderated the effects of stimulation. Chronotype was assessed using the German version of the Morningness–Eveningness questionnaire (D-MEQ) (Griefahn et al., 2001). We considered the MEQ score as a continuous and as a categorical covariate (two groups: morning vs. intermediate/late type). Given our older adult sample (age 55–79 years, mean 66 years), the distribution of chronotypes was strongly skewed toward morningness, consisting of *n* = 12 morning types (*n* = 3 definite, *n* = 9 moderate), *n* = 9 intermediate types, and only *n* = 1 moderate evening type. This age-related shift toward morningness is well-documented in the literature, as chronotype remains relatively stable in adulthood but shows a pronounced shift toward earlier chronotypes with advancing age (Roenneberg et al., 2007; Adan et al., 2012). Therefore, we dichotomized our sample into morning types versus intermediate- to evening-types to enable meaningful statistical comparisons. The cutoff was determined according to the D-MEQ scoring manual: scores of 42–58 were classified as intermediate/evening types, and scores of 59 and above as morning types (Griefahn et al., 2001).

### Statistical analyses

All statistical analyses were performed using Python (NumPy, pandas, pycircstat2) and SPSS (Version 29.0). Unless indicated otherwise, statistical significance was defined as *p* < 0.05, with multiple comparisons for planned contrasts corrected using the Holm-Bonferroni method to control familywise error. SO event characteristics, spectral power in SO and spindle frequency bands, SO–spindle coupling strength, and E/I ratio (extracted for 1-min post-stimulation intervals) were compared across conditions using repeated-measures ANOVA. When omnibus effects were significant, planned contrasts were conducted comparing cathodal versus sham and anodal versus sham stimulation. For TFR analyses, a two-tailed paired-samples *t*-test was conducted to determine statistical significance between the conditions at the group level. Next, cluster-based permutation testing was applied to correct for multiple comparisons (see TFR section for details).

Chronotype moderation was assessed using two approaches. First, morning–evening preference (MEQ score) was entered as a continuous moderator in a repeated-measures ANCOVA, with moderation tested via the Condition × MEQ interaction. Second, categorical chronotype groups were additionally compared using a mixed-model ANOVA testing the Condition × Chronotype interaction, enabling direct between-group comparisons.

Additional exploratory analyses assessed associations between individual (continuous) chronotype and NREM sleep parameter as well as between spectral slope, spindle activity, and SO-spindle coupling. These regression analyses of spectral slope and additional exploratory comparisons (e.g., sleep macrostructure) were designated as exploratory, and *p*-values should be interpreted within this framework.

Sample size estimation was performed using G*Power, informed by prior research on anodal so-tDCS during NREM (Ladenbauer et al., 2017). While that study observed large effect sizes (*d* > 0.7) on SO and spindle activity, we adopted a more conservative estimate of *d* = 0.55 given the small sample (*n* = 16) and the absence of prior cathodal NREM studies targeting these outcomes. With *α* = 0.05 (two-tailed paired *t*-test) and *n* = 22, this yields approximately 80% power to detect stimulation effects.

## Results

### Cathodal so-tDCS effects

**Figure 2:**
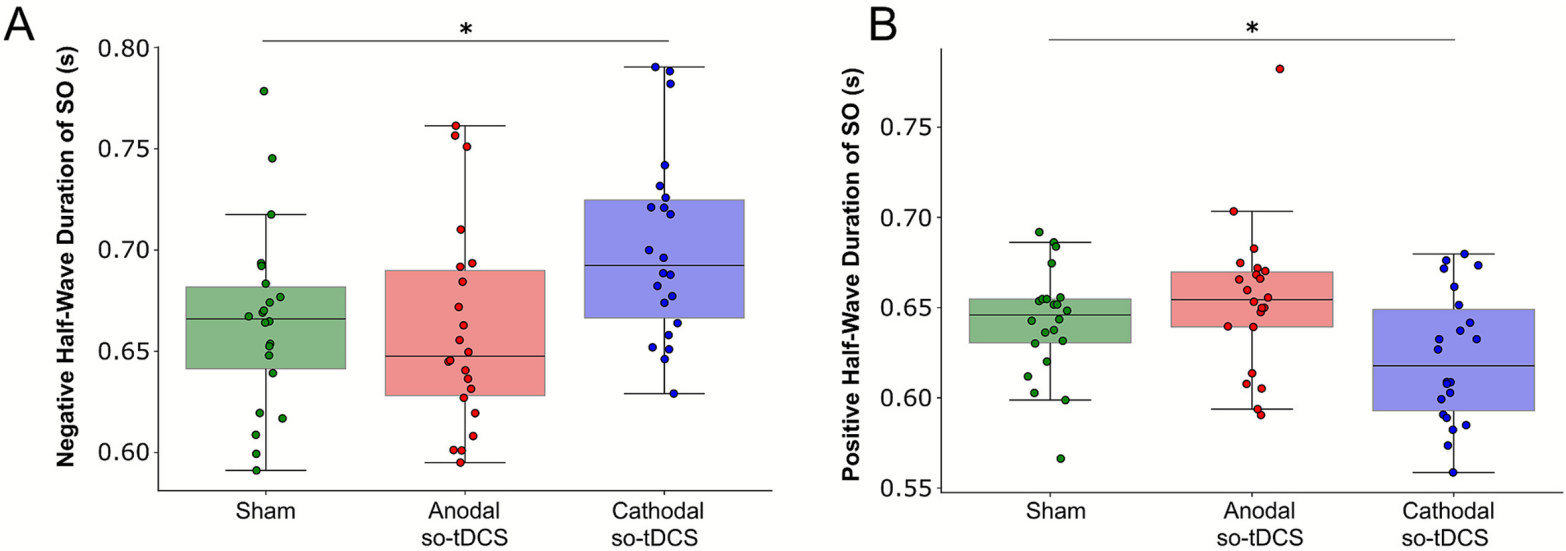
Cathodal so-tDCS modulates SO durations. **(A)** Negative half-wave and **(B)** positive half-wave duration of SO by condition. Cathodal so-tDCS prolonged negative half-wave durations of SO and shortened positive half-wave durations of SO relative to sham. *p < 0.05.

We first focused on the effects of cathodal so-tDCS on the SO parameter. Cathodal so-tDCS did not influence the primary SO amplitude measure in this evaluation: the magnitude of SO down-states (*F*_(2, 42)_ = 0.961, *p* = 0.37, Greenhouse–Geisser corrected). Furthermore, no significant changes were observed in SO count, SO up-state amplitudes, slope of SO events, or the overall peak-to-peak SO amplitudes relative to sham (all *p*-values > 0.1; see Supplementary Table S2 for results of all outcome measures). Similarly, spectral SO power remained unaffected by cathodal so-tDCS (F_(2, 42)_ = 1.419, *p* = 0.25). However, cathodal so-tDCS strongly affected the *negative half-wave duration of* SO periods, resulting in prolonged *negative half-wave SO durations* (*F*_(2, 42)_ = 6.132, *p* = 0.003, Holm–Bonferroni adjusted *p*-value: cathodal-sham: *p*-adj = 0.015, Cohen’s *d* = 0.668, Figure 2A), indicating a prolonged hyperpolarizing phase of the SO cycle. In addition, the positive half-wave duration of SO was shortened after cathodal so-tDCS relative to sham (*F*_(2, 42)_ = 6.432, *p* = 0.005; anodal-sham p-adj = 0.032, Cohen’s *d* = 0.56, Figure 2B).

**Figure 3:**
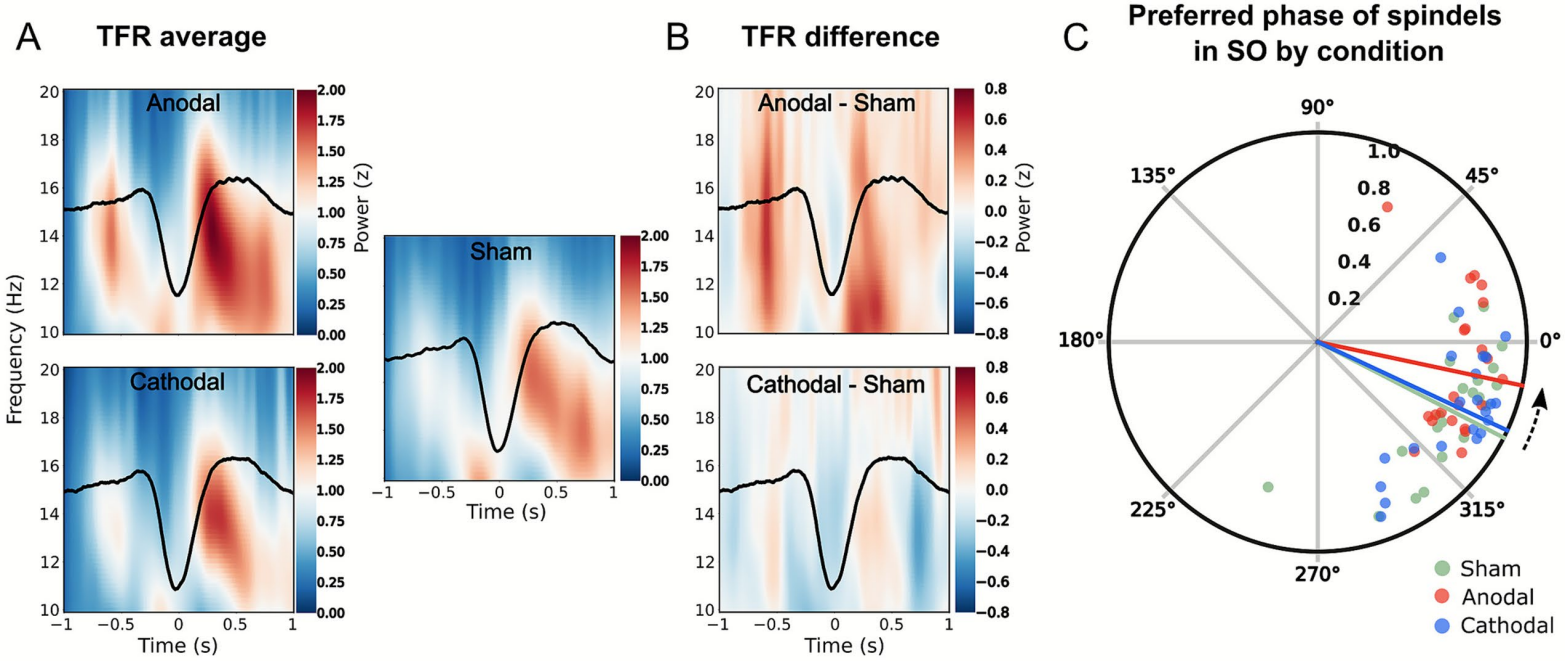
Stimulation polarity impacts on slow oscillation (SO)-spindle coupling. **(A)** Time-frequency representations (TFRs) of SO-locked power, averaged across subjects for each stimulation condition, shown as a change from pre-event baseline. **(B)** Statistical map of condition differences in SO-locked power. Note that spindle-band (12–18 Hz) activity during SO up-phases increases in the anodal condition, but not in the cathodal condition, relative to sham. **(C)** Polar plot showing the mean SO phase at which spindle power peaks for each condition (colored lines), with individual subject averages represented by dots for each condition: sham (green), anodal (red), and cathodal (blue). Note that spindle timing in the anodal so-tDCS condition is shifted toward the SO up-state (0°), indicating enhanced phase synchrony compared to sham and cathodal so-tDCS.

In terms of sleep spindle activity, cathodal so-tDCS did not significantly alter spindle power relative to sham (*F*_(2, 42)_ = 1.554, *p* = 0.223). Furthermore, no significant effects of cathodal so-tDCS were observed on SO-spindle coupling. Specifically, it did not increase spindle activity during the SO upstate in the TFR analysis relative to sham (see Figure 3), and circular analyses of mean coupling direction of spindles within the SO cycle showed only minimal differences compared to sham (circular Watson-Williams test: *p* = 0.794; Cohen’s *d* = 0.1208, mean phase difference = 2.21° [95% CI: −13.47° to 17.89°]), indicating a negligible overall cathodal so-tDCS effect on temporal synchrony between SO and spindle activity. Consistent with these findings, cathodal so-tDCS showed no impact on SO-spindle coupling strength (*F*_(2, 42)_ = 0.020, *p* = 0.98).

To assess effects on cortical excitability, the slope of the EEG power spectral density was used as a proxy measure of the E/I balance (Gao et al., 2017). Although a slight increase in spectral slope was observed under cathodal so-tDCS relative to sham, this change failed to reach statistical significance (*F*_(2, 42)_ = 6.121, *p* = 0.005, partial *η*^2^ = 0.226; planed contrast cathodal vs. sham: *p*-adj = 0.073; Cohen’s *d* = 0.402).

### Anodal so-tDCS effects

Anodal oscillatory tDCS demonstrated a markedly different pattern of effects across the same sleep parameters. Parallel analyses for anodal so-tDCS revealed similarly negligible effects on SO parameters. Specifically, no significant alterations were observed in the magnitude of SO down-states, SO up-states, number of SO events, SO slope, peak-to-peak amplitude, or duration of positive half-wave and negative half-wave duration of the SO events compared to sham (all *p* > 0.1; see Supplementary Table S2 for results of all outcome measures). Similarly, SO and spindle power remained unaffected by anodal so-tDCS (*F*_(2, 42)_ = 1.419, *p* = 0.25; and *F*_(2, 42)_ = 1.554, *p* = 0.223, respectively). However, anodal so-tDCS facilitated SO-spindle coupling. Time-frequency representations indicated enhanced spindle power during the late rising phase of the SO following anodal so-tDCS relative to sham (see Figures 3A,B). Although the SO-epoch interval did not reach statistical significance after a cluster-based permutation test to account for multiple comparisons, the trend clearly indicates an increase in spindle activity during the depolarizing SO up-phase. Complementary circular statistical analyses confirmed a strong shift in the preferred spindle phase after anodal so-tDCS compared to sham, with spindles occurring closer to the SO upstate peak (mean angle ± SD: anodal = 348.0° ± 26.25°; sham = 332.84° ± 27.24°), indicating improved precision of spindle alignment relative to sham (Figure 3C). The mean difference (15.16°; 95% CI: −0.65° to 30.97°) indicated a large effect size (Cohen’s *d* = 0.824), though the difference marginally missed statistical significance (Watson–Williams test: *p* = 0.081). Coupling strength remained unchanged (*F*_(2, 42)_ = 0.020, *p* = 0.98), suggesting that the effect of anodal so-tDCS relative to sham was specific to timing, rather than general coupling strength.

**Figure 4:**
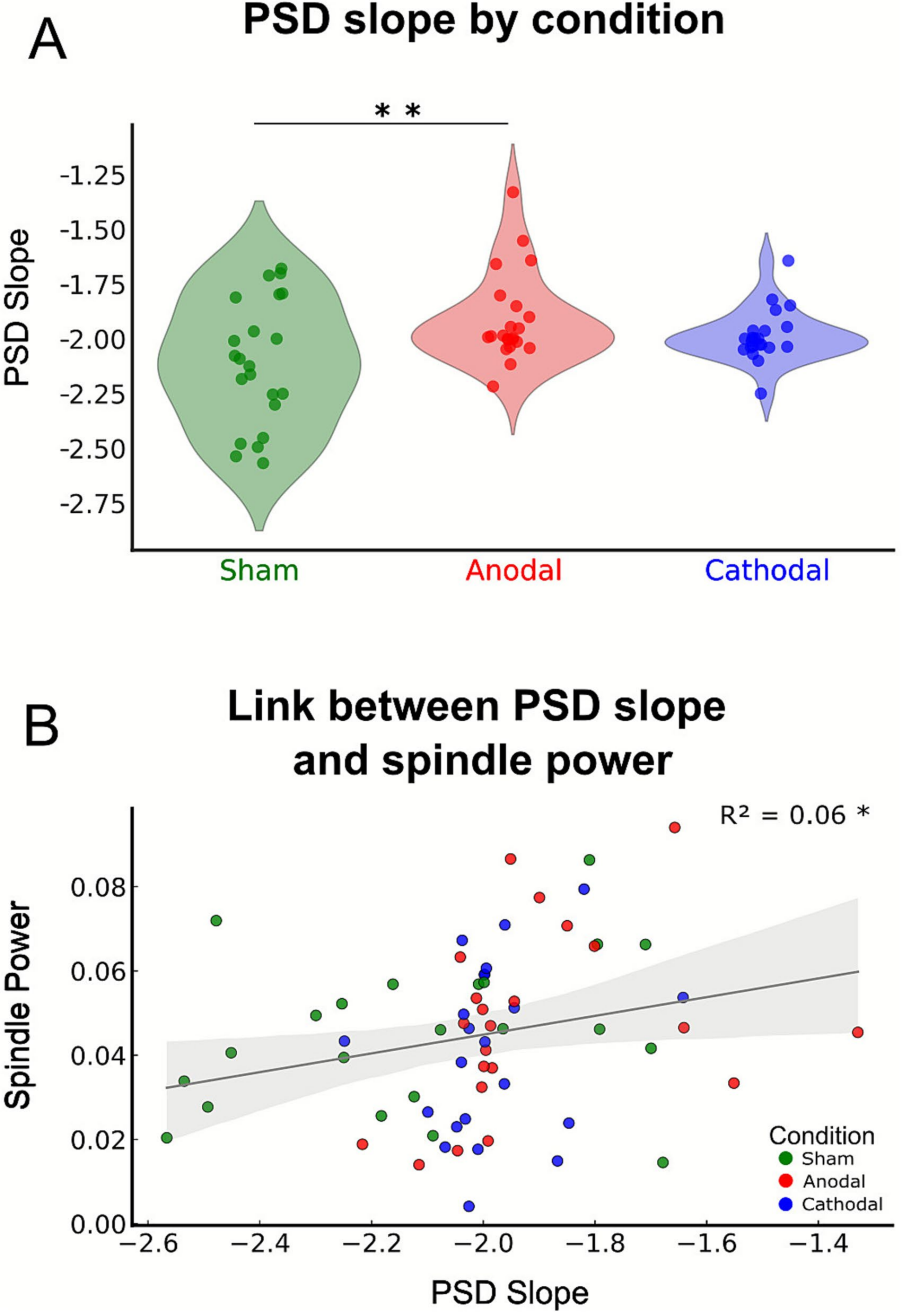
so-tDCS effects on excitation/inhibition (E/I) balance and PSD slope association with spindle power. **(A)** Power spectral density (PSD) slope across conditions. Note that PSD slope is increased (less negative) after anodal so-tDCS relative to sham. Cathodal so-tDCS shows a similar trend. **(B)** Positive association between PSD slope and spindle power across conditions, suggesting that E/I balance contributes to spindle power expression, though with very modest explanatory variance. *p < 0.05, **p < 0.01.

Further significant results emerged in the context of cortical excitability, where anodal so-tDCS produced a robust shift toward greater excitation in E/I balance relative to sham (*F*_(2, 42)_ = 6.121, *p* = 0.005; p-adj = 0.004, Cohen’s *d* = 0.752, see Figure 4), indicating enhanced cortical excitability. Baseline spectral slopes prior to stimulation onset did not differ between conditions (*F*_(2, 42)_ = 0.226, *p* = 0.743).

A comparative evaluation highlighted polarity-specific effects of so-tDCS on NREM sleep microstructure. While only cathodal so-tDCS influenced SO durations, with prolonged negative half-wave of SO phases and shortened positive half-wave of SO phases, anodal so-tDCS markedly promoted SO-spindle coupling, evident by increased spindle activity during the SO up-state as well as improved synchrony of SO-spindle coupling. In addition, anodal so-tDCS markedly shifted E/I balance in the direction of increased excitability. In contrast, cathodal so-tDCS failed to produce significant effects on these outcomes, although there was a trend in the same direction, especially for E/I balance.

### Morning-evening preference as a moderator

**Figure 5:**
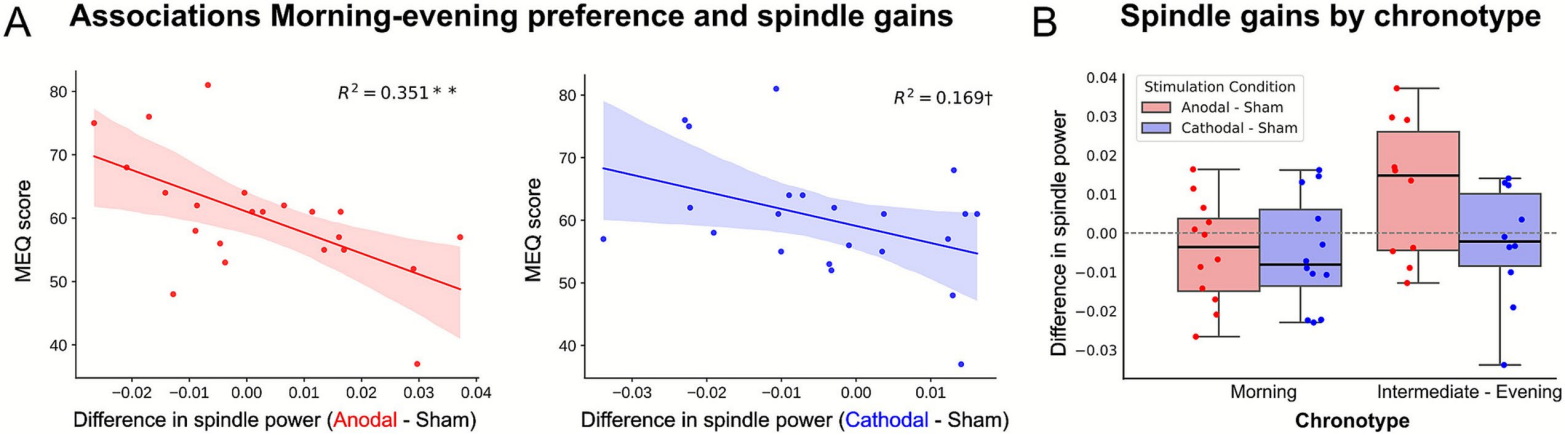
Chronotype moderates the effects of so-tDCS on spindle activity. **(A)** Spindle gains in anodal (left) and cathodal so-tDCS (right) conditions plotted against individual MEQ scores. Spindle power changes after anodal so-tDCS are strongest for intermediate- and evening preference (MEQ score < 59) relative to sham. A similar, though much weaker, trend is observed following cathodal so-tDCS. **(B)** Spindle power changes after anodal (red) and cathodal (blue) so-tDCS (relative to sham) displayed by chronotype group. **p < 0.01. ^†^*p* < 0.1.

Chronotype, specifically the continuous degree of preference for morning or evening activity (i.e., MEQ score), emerged as an important moderator of anodal so-tDCS effects on spindle activity. We observed an interaction effect between stimulation condition and morning-evening preference (i.e., MEQ score; *F*_(2, 40)_ = 4.066, *p* = 0.038, Greenhouse–Geisser corrected, partial *η*^2^ = 0.169). Individuals with low MEQ score, classified as intermediate-to-evening types (MEQ score <59; *n* = 10), showed increases in spindle power after anodal so-tDCS compared to sham (stimulation condition x chronotype group: *F*_(2, 40)_ = 2.87, *p* = 0.068, partial *η*^2^ = 0.13; mean change ± SD for individuals with low MEQ score: 24.1% ± 72.1%, pairwise comparison *p* = 0.077; see Supplementary Table S3). Conversely, morning-types (*n* = 12) experienced no spindle power benefit or decrease following anodal so-tDCS (mean change ± SD: −10.6% ± 54.5%, pairwise comparison *p* = 0.246; see Figure 5B). By comparison in cathodal condition, neither intermediate-to-evening nor morning types showed effects on spindle power compared to sham (mean change ± SD: −6.01% ± 57.88%, *p* = 0.575; and −11.32% ± 60.70%, *p* = 0.250, respectively; see Figure 5B). This finding was substantiated by a strong negative correlation between MEQ scores and individual anodal so-tDCS-induced spindle increase (*r* = −0.59, *p* = 0.004), with MEQ scores explaining 35.1% of the variance in spindle power change after anodal so-tDCS compared to sham (see Figure 5A). For cathodal so-tDCS, the negative correlation with MEQ score demonstrated a smaller effect size that approached but did not reach statistical significance (*r* = −0.41, *p* = 0.057, *R*^2^ = 0.169), indicating a similar directional relationship but with much weaker and less consistent chronotype modulation effect compared to anodal so-tDCS (see Figure 5A). No other interaction effect with chronotype was evident for all other investigated NREM sleep parameter (all *p* > 0.3).

Intervals between morning wake-up and nap onsets differed by chronotype, as expected. Mean Intervals between morning wake-up and nap onset were 8.01 ± 1.30 h for morning chronotypes and 6.96 ± 0.71 h for intermediate-to-evening chronotypes, resulting in a significant difference between chronotype groups (*p* < 0.034). There were no differences in wake-up times (and intervals) between experimental conditions (*p* = 0.533), as participants maintained consistent sleep–wake schedules across sessions (see Supplementary Table S4).

Given the robust effects of anodal so-tDCS on spectral slope observed in our data, and prior evidence for the role of excitation/inhibition balance in shaping cortical network responsiveness and oscillatory dynamics (Haider and McCormick, 2009), we additionally examined whether spectral slope was predictive for slow oscillation and spindle power, as well as SO-spindle coupling, across stimulation conditions. These regression analyses revealed that E/I balance significantly predicted spindle power (*B* = 0.022, *β* = 0.25, SE = 0.11, *t* = 2.064, *p* = 0.043, Figure 4B), though with very modest explanatory variance (6.2%), while it showed no relationship with coupling phase (*p* = 0.289, *R*^2^ = 0.018). In addition to our primary objective of examining the impact of so-tDCS on sleep microstructure, we evaluated whether so-tDCS conditions influenced macro-sleep architecture, including sleep-stage proportions (across the entire nap and within 1-min post-stimulation intervals), sleep efficiency, and sleep-stage latencies. Cathodal so-tDCS had no significant effects on any of these macro-sleep parameters compared to sham stimulation (all *p* > 0.1; see Supplementary Table S5). Conversely, during anodal so-tDCS, we observed a reduced proportion of slow-wave sleep (N3), along with an increased proportion of lighter sleep (N1), suggesting a shift toward shallower sleep under anodal so-tDCS. However, this effect may partly reflect in part pre-stimulation differences, as latencies to N2 (and consequently N3) sleep onset were longer during the anodal condition, thereby biasing subsequent sleep-stage proportions. Supporting this interpretation, analyses of 1-min post-stimulation intervals immediately following so-tDCS/sham blocks revealed no significant differences between conditions (all *p* > 0.1; see Supplementary Table S5). Notably, a trend emerged toward fewer brief awakenings (WASO) in post-stimulation intervals following anodal so-tDCS as compared to sham (*p* = 0.062), indicating that anodal so-tDCS may exert protective effects against awakenings. Nevertheless, these results collectively suggest that anodal so-tDCS may preferentially support N2 rather than deeper N3 sleep stages. Furthermore, the use of a state-dependent stimulus on a protocol requiring N2/N3 sleep may have introduced variability in the number of stimulation and sham blocks across conditions. However, the number of blocks did not differ significantly among the three conditions (Friedman test: *χ*^2^(2) = 3.534, *p* = 0.171; see Supplementary Table S6 for the number of stimulation blocks per condition).

In sum, cathodal and anodal so-tDCS elicited distinct and polarity-specific effects on sleep-related neural oscillations. While cathodal so-tDCS influenced SO durations, it did not significantly affect other sleep parameters. In contrast, anodal so-tDCS promoted more precise SO-spindle phase alignment, increased cortical excitability, and enhanced spindle power in individuals with an intermediate- to evening-chronotype.

## Discussion

In the present study, we investigated the impact of so-tDCS polarity on the dynamics of NREM sleep oscillations that support memory consolidation in healthy older adults. We focused on how cathodal so-tDCS modulates cortical SO and thalamocortical spindle activity, and their phase coupling, compared these outcomes to anodal so-tDCS effects (both relative to sham), and determined the influence of chronotype on individual responses to both so-tDCS protocols. Our findings reveal a clear polarity-dependent difference: cathodal so-tDCS prolonged SO down-states and shortened SO up-states, which may reflect amplified cortical hyperpolarization, but did not impact SO down-state amplitude, spindle activity, nor SO-spindle coupling. In contrast, anodal so-tDCS induced a marked shift in spindle timing toward the SO up-state, consistent with tighter and thus improved SO-spindle coupling, and led to a marked increase in cortical excitability compared to sham. Furthermore, individual chronotype emerged as a key modulator: participants with a later chronotype showed the strongest anodal-induced increases in spindle activity.

### Cathodal so-tDCS prolonged down-state without spindle enhancement

Based on Mak-McCully et al. (2017), we hypothesized that cathodal so-tDCS, by hyperpolarizing cortical neurons (Nitsche et al., 2003) during the down-state, would increase down-states as compared to sham, and thereby influence spindle generation. We found that cathodal so-tDCS indeed prolonged the duration of the SO down-state, which may reflect an augmented hyperpolarized state that, in turn, may allow greater thalamic deactivation and a more pronounced rebound spindle burst, according to the mechanism proposed by Mak-McCully et al. (2017). However, in our experimental setting, cathodal so-tDCS did not lead to changes in spindle power, nor in the precise timing of spindles relative to the SO cycle. This failure to modulate spindle activity could be attributed to several factors. First, while evidence indicates that synchronized cortical down-states can influence thalamic rebound spindles (Mak-McCully et al., 2017), it is likely that an optimal timing and dynamic between down- and up-states is required for effective spindle initiation (Niethard et al., 2018; Bazhenov et al., 2002; Mushtaq et al., 2024). Second, prolonging the down-state might not enhance this temporal pattern, possibly even disrupt it (Bazhenov et al., 2002). Third, during natural NREM sleep, cortical neurons are already hyperpolarized during down-states (Steriade, 2001), potentially limiting the additional impact of externally applied hyperpolarizing currents on the amplitude of the down-state. In this scenario, cathodal so-tDCS might offer little additive modulation due to a ceiling effect in membrane hyperpolarization.

It is also notable that we did not observe a decrease in the EEG spectral slope under cathodal so-tDCS. The spectral slope is increasingly recognized as a proxy for cortical excitability, with a more negative (steeper) slope reflecting stronger relative inhibition (Gao et al., 2017). The lack of spectral slope steepening suggests that cathodal so-tDCS did not meaningfully shift the E/I balance toward inhibition. In fact, the direction of change was toward slight flattening, indicating a trendwise increase in excitability. These findings are consistent with a growing body of evidence showing that cathodal tDCS does not reliably produce the expected hyperpolarizing effects (Tanaka et al., 2013; Tanaka et al., 2020; Mosayebi-Samani et al., 2023).

### Anodal so-tDCS modulated SO-spindle coupling and increased cortical excitability

Anodal so-tDCS showed indications of beneficially modulating NREM sleep oscillations. The descriptive pattern was broadly consistent with prior work reporting anodal so-tDCS-related increases in spindle activity during SO events (Ladenbauer et al., 2017; Ladenbauer et al., 2023), but this trend did not survive cluster-based permutation testing in the current experiment. Spindles were also more precisely timed within SO events: Under anodal oscillatory tDCS, compared to sham, spindles occurred closer to the peak of the SO up-state. Although this phase shift did not reach statistical significance, the large effect size supports the interpretation that it may reflect a meaningful tightening of SO-spindle coupling, a parameter known as critical for effective memory consolidation, and typically more temporally dispersed in older adults (Helfrich et al., 2018; Züst et al., 2023). Thus, while cathodal so-tDCS primarily affected the duration of SO components, without clearly engaging downstream oscillatory processes, anodal so-tDCS may have interacted more directly with thalamocortical processes implicated in memory consolidation. These distinctions point to polarity as a key determinant of how so-tDCS modulates sleep-related neural dynamics.

Anodal so-tDCS also shifted E/I balance toward increased excitability during NREM sleep, i.e., increased cortical excitability. This finding aligns well with previous reports demonstrating that continuous anodal tDCS reliably enhances cortical excitability during wakefulness (Bastani and Jaberzadeh, 2012).

Mechanistically, the elevated cortical excitability induced by anodal so-tDCS may have contributed to improving SO-spindle coupling and enhanced spindle activity during SO up-states. These effects likely reflect increased synaptic responsiveness, consistent with the “excitable Up-state regime” of NREM sleep proposed by Levenstein et al. (2019). Using combined *in vivo* recordings and computational modeling, they demonstrated that the neocortex during NREM sleep is predominantly in an active Up-state, which is characterized by sustained neuronal firing and only transient Down-states. These findings challenged earlier conceptions of NREM sleep as a period of globally reduced neural activity and responsiveness (Steriade and Amzica, 1998), suggesting instead a dynamic, high-excitability cortical network in which SO down-states can arise from minor fluctuations in cortical activity. Therefore, by promoting this excitable Up-state regime during NREM sleep and potentially amplifying rhythmic fluctuations via the oscillatory nature of the stimulation, anodal so-tDCS may have improved SO-spindle coupling.

The induced rise in cortical excitability, nevertheless, raises important questions regarding its interaction with sleep homeostasis. Sleep was shown to downscale synaptic weights and normalize excitability accumulated during wakefulness (Tononi and Cirelli, 2006). Externally increasing excitability during NREM sleep could, in theory, interfere with this restorative process. Although we did not assess E/I balance before and after sleep, compensatory mechanisms during subsequent phases, such as REM sleep, may help restore network homeostasis. Indeed, recent evidence suggests that REM sleep can recalibrate cortical excitability and supports neural homeostasis (Lendner et al., 2023). Future studies should determine whether anodal so-tDCS-induced excitability increase during NREM sleep is balanced by a compensatory response in later sleep stages.

Taken together, polarity of so-tDCS was a critical determinant of its functional efficacy, with anodal so-tDCS demonstrating superior impact on sleep microstructure relevant to memory.

### Chronotype modulated spindle response to anodal so-tDCS

Impact of so-tDCS on oscillatory activity has varied across individuals (Ladenbauer et al., 2017) and studies (Barham et al., 2016), likely due to differences in methodology and participant characteristics. Recent findings indicated that an individual’s circadian preference for morning or evening activity influences baseline cortical excitability and the responsiveness to continuous tDCS-induced neuroplasticity during wakefulness (Salehinejad et al., 2021). We therefore assessed chronotype as a potential moderator for so-tDCS during sleep and found that it strongly influenced the efficacy of anodal so-tDCS in enhancing spindle activity. While cathodal so-tDCS followed the same direction, the effect size was small. Importantly, chronotype groups did not differ in any baseline sleep measures or in cortical E/I balance prior to stimulation, indicating that both morning- and intermediate-to-evening groups were comparable in sleep oscillation characteristics and cortical excitability during NREM sleep prior to stimulation onset. The differential responsiveness emerged only after so-tDCS onset, suggesting that chronotype is linked to the brain’s capacity to undergo plastic changes induced by so-tDCS, rather than to intrinsic differences in sleep timing.

These results align with and extend recent findings from Salehinejad et al. (2021), who reported that individuals exhibit greater neuroplastic changes following continuous tDCS during wakefulness if stimulation was applied at their “preferred” circadian time. In our nap paradigm, anodal so-tDCS was most effective in intermediate- to moderate evening-types, possibly because our stimulation timing (early afternoon) better coincided with their optimal neuroplastic window, i.e., peak of alertness and neural responsiveness.

Future studies should determine whether these nap-derived effects generalize to nighttime sleep, where a stronger homeostatic sleep drive and different circadian phases may alter stimulation responsiveness. Moreover, it should be assessed whether subsequent REM sleep compensates for anodal-induced excitability increases to maintain neural homeostasis.

## Limitations

Two limitations should be acknowledged. First, the sample size (*N* = 22) was modest, which may limit the generalizability of our findings and the power to detect more subtle effects. While our within-subject design enhances statistical sensitivity, a larger sample would allow more nuanced analysis of subgroup effects, particularly for chronotype. The strong correlation between chronotype and spindle enhancement suggests this factor warrants further study in larger, more diverse cohorts (i.e., including more evening chronotype individuals). Second, the cathodal condition, designed to compare the neurophysiological effects of cathodal so-tDCS in an amendment to the original protocol, did not include a memory task before or after the nap. This may represent a potential confound, as pre-sleep learning has been shown to enhance subsequent SO and spindle activity (Gais et al., 2002), potentially biasing conditions involving pre-nap learning toward stronger oscillatory responses during sleep. However, we consider it unlikely that this influenced our findings for several reasons: (i) no main effect of condition (anodal with memory task vs. cathodal without memory task) relative to sham was observed on spindle or SO activity, and baseline N2 sleep prior to stimulation onset showed no differences in SO or spindle power, their coupling, or E/I balance across conditions; (ii) individual differences in chronotype, rather than condition and prior learning, emerged as the primary modulator of stimulation responsiveness on spindle activity, with spindle enhancement being evident primarily in participants with neutral to late chronotype tested during their preferred time of day; and (iii) the effects of specific outcome measures in our study, E/I balance and SO-spindle coupling phase, have been demonstrated to be learning-independent in prior research (Cox et al., 2018; Tamaki et al., 2020).

As expected, intermediate-to-evening chronotypes had significantly shorter wake durations before the nap session compared to morning chronotypes. Because nap timing was fixed by clock time, this likely resulted in differences in circadian timing at nap onset as well as differences in homeostatic sleep pressure. This systematic variation in physiological state at nap onset may have contributed to the observed chronotype-dependent effects. Without pre-nap resting EEG data to assess sleep pressure markers (e.g., theta power), we cannot determine whether chronotype differences in so-tDCS responsiveness reflect accumulated sleep pressure or intrinsic neurophysiological differences. To address this, future studies should incorporate pre-nap EEG assessments or control wake duration across individuals and consider scheduling naps relative to each participant’s circadian phase to reduce variability in both sleep pressure and circadian timing.

## Conclusion

In conclusion, our findings demonstrate that anodal so-tDCS during NREM sleep enhances thalamocortical oscillatory mechanisms associated with memory consolidation in older adults, whereas cathodal so-tDCS, despite modulating SO morphology, fails to engage these functional pathways and offers no observable benefit. These findings further support the concept of an excitable cortical UP-state during NREM sleep and highlight the moderating role of individual traits such as chronotype in brain stimulation protocols during sleep, emphasizing the need for personalized stimulation protocols. Furthermore, from a translational standpoint, such knowledge could contribute to interventions for cognitive aging and Alzheimer’s disease, where improving sleep oscillation quality holds therapeutic promise (Ladenbauer et al., 2017; Chylinski et al., 2022).

## Data Availability

The datasets presented in this article are not readily available because of privacy and ethical restrictions related to the study’s participants. Requests to access the datasets should be directed to Julia Ladenbauer, Julia.Ladenbauer@med.uni-greifswald.de and Agnes Flöel, Agnes.Floeel@med.uni-greifswald.de.

## Footnotes

1
https://github.com/jshanna100/sfb/tree/master/sfb2

2
https://github.com/jbkordass/memory-sfb2-2025
