# Supplementary material for: Polarity-dependent modulation of sleep oscillations and cortical excitability in aging
Source: Front Aging Neurosci. 2026 Jan 15;17:1704130. doi: 10.3389/fnagi.2025.1704130 (PMC12852367; doi:10.3389/fnagi.2025.1704130)
Supplement: Supplementary file 1 [file Table_1.pdf]

Table S1. Baseline characteristics

n (female/male)	22 (10/12)
Age (y)	66.4 ± 6.7 (55-79)
Education duration (y)	14.0 ± 3.4 (4-20) *
D-MEQ	60.2 ± 9.5 (37-81)
AVLT, learning	54.5 ± 9.3 (35-70)
AVLT, delayed recall	1.7 ± 1.9 (-2-6)
Digit span, forward	6.7 ± 2.1 (2-11)
Digit span, backwards	6.0 ± 1.7 (2-8)
TMT, part A (time to complete,s)	33.6 ± 8.5 (18-55)
TMT, part B (time to complete,s)	78.2 ± 21.8 (41-121)
Letter Digit Substitution	29.5 ± 4.8 (23-41)
Constructional Praxis, copy	10.5 ± 1.1 (8-12)
Constructional Praxis, delayed recall	9.0 ± 4.8 (4-28)
Mini-Mental-State Examination	27.5 ± 4.7 (8-30)
Stroop color-word test (delay for incongruent vs neutral condition)	98.3 ± 21.0 (72-158)
Verbal fluency, phonematic (no. of words)	13.9 ± 4.2 (7-25)
Verbal fluency, semantic (no. of words)	22.4 ± 5.1 (11-31)
Multiple choice vocabulary intelligence test	31.7 ± 2.4 (26-35)

Data are given as mean ± SD and range (minimum to maximum).
AVLT = auditory verbal learning test.
D-MEQ = German version of Morning-Evening-Questionnaire.
*Data available for 20/22 participants due to missing values.
