# Supplementary material for: Polarity-dependent modulation of sleep oscillations and cortical excitability in aging
Source: Front Aging Neurosci. 2026 Jan 15;17:1704130. doi: 10.3389/fnagi.2025.1704130 (PMC12852367; doi:10.3389/fnagi.2025.1704130)
Supplement: Supplementary file 2 [file Table_2.pdf]

Table S2. All outcomes of sleep microstructure and E/I balance per condition

Event type and feature	Cathodal so-tDCS mean (SD)	Anodal so-tDCS mean (SD)	Sham mean (SD)	p-value
**Slow Oscillation (SO) Parameters**				
SO trough amplitude (in µV)	-44.558 (63.435)	-25.355 (8.791)	-40.453 (54.845)	0.371#
SO peak amplitude (in µV)	39.754 (65.654)	25.728 (9.097)	35.794 (46.197)	0.537#
Peak-to-Peak Amplitude (in µV)	84.312 (128.887)	51.083 (17.235)	76.247 (100.927)	0.385##
SO slope (in µV/s)	157.94 (198.95)	104.79 (40.57)	153.05 (195.36)	0.244##
positive half-wave SO duration (in s)	0.613 (0.037)	0.658 (0.049)	0.641 (0.039)	0.005**
negative half-wave SO duration (in s)	0.712 (0.012)	0.659 (0.015)	0.657 (0.107)	0.003**
Number of SO events (N)	147.45 (92.042)	135.32 (81.502)	184.50 (82.331)	0.463##
SO-power (in µV)	0.294 (0.088)	0.278 (0.072)	0.275 (0.075)	0.253
**Spindle Activity and Coupling to SO events**				
Spindle power (in µV)	0.041(0.020)	0.048 (0.022)	0.045 (0.018)	0.223
Mean direction of SO-spindle coupling (in °)	335.0 ° ± 25.82°	348.0° ± 26.25°	332.84° ± 27.24°	0.081 (anodal-sham)<br>0.794 (cathodal-sham)
Coupling strength of SO-spindle coupling	0.214 (0.063)	0.218 (0.101)	0.218 (0.075)	0.980
**E/I balance**				
PSD slope (aperiodic exponent)	-1.986 (0.117)	-1.912 (0.203)	-2.110 (0.286)	0.005**
Baseline spectral slope (aperiodic exponent)	-2.008 (0.281)	-1.960 (0.395)	-2.020 (0.325)	0.743#

*denotes p<0.05, ** p<0.01, #Greenhouse-Geisser corrected, ##Friedman test
