# Supplementary material for: Polarity-dependent modulation of sleep oscillations and cortical excitability in aging
Source: Front Aging Neurosci. 2026 Jan 15;17:1704130. doi: 10.3389/fnagi.2025.1704130 (PMC12852367; doi:10.3389/fnagi.2025.1704130)
Supplement: Supplementary file 3 [file Table_3.pdf]

Table S3. Main and interaction effects of stimulation with categorical chronotype (2 groups)

Effect	F (df)	p-value	η^2p
**Spindle power (12-15 Hz)**			
Stimulation condition	F(2, 40) = 1.99	0.151	0.09
Chronotype (dichotomized)	F(1, 20) = 1.29	0.269	0.06
Stimulation × Chronotype	F(2, 40) = 2.87	0.068	0.13
**SO power (0.5-1 Hz)**			
Stimulation condition	F(2, 40) = 1.57	0.221	0.07
Chronotype (dichotomized)	F(1, 20) = 0.56	0.464	0.03
Stimulation × Chronotype	F(2, 40) = 1.42	0.253	0.07
**Coupling strength of SO-spindle coupling**			
Stimulation condition	F(2, 40) = 0.04	0.963	0.00
Chronotype (dichotomized)	F(1, 20) = 3.66	0.070	0.16
Stimulation × Chronotype	F(2, 40) = 0.37	0.695	0.02
**E/I balance (PSD slope)**			
Stimulation condition	F(2, 40) = 0.27	0.271[#]	0.01
Chronotype (dichotomized)	F(1, 20) = 2.29	0.146	0.10
Stimulation × Chronotype	F(2, 40) = 0.40	0.623[#]	0.02

[#]Greenhouse-Geisser corrected
