# Supplementary material for: Polarity-dependent modulation of sleep oscillations and cortical excitability in aging
Source: Front Aging Neurosci. 2026 Jan 15;17:1704130. doi: 10.3389/fnagi.2025.1704130 (PMC12852367; doi:10.3389/fnagi.2025.1704130)
Supplement: Supplementary file 4 [file Table_4.pdf]

Table S4. Comparisons of awake time and duration of wakefulness between chronotype groups and conditions

Variable	Awake time (decimal hrs) (mean ± SD)	Duration of wakefulness (decimal hrs) (mean ± SD)	p-value
**Chronotype**			
Morning chronotype (n = 12)	5.99 ± 1.30	8.01 ± 1.30	0.034*
Intermediate-to-evening chronotype (n = 10)	7.04 ± 0.71	6.96 ± 0.71	
**Condition**			
Cathodal so-tDCS	6.66 ± 1.30	7.34 ± 1.30	0.533
Anodal so-tDCS	6.43 ± 1.34	7.57 ± 1.34	
Sham	6.41 ± 1.39	7.59 ± 1.39	

Data are given as mean ± SD and range; SD = standard deviation
Duration of wakefulness = time interval between awake time and nap onset
Morning chronotype = definitive morning type (n = 3) + moderate morning type (n = 9)
Intermediate-to-evening chronotype = moderate evening type (n = 1) + intermediate type (n = 9)
*denotes $p<0.05$
