# Supplementary material for: Polarity-dependent modulation of sleep oscillations and cortical excitability in aging
Source: Front Aging Neurosci. 2026 Jan 15;17:1704130. doi: 10.3389/fnagi.2025.1704130 (PMC12852367; doi:10.3389/fnagi.2025.1704130)
Supplement: Supplementary file 5 [file Table_5.pdf]

Table S5: Sleep architecture

	cathodal so-tDCS		anodal so-tDCS		sham		cathodal vs. sham		anodal vs. sham
	**Mean (SD)**	**MD**	**Mean (SD)**	**MD**	**Mean (SD)**	**MD**	**p**	**corr. p**	**corr. p**
**Sleep architecture in 1-minute post-stimulation intervals**									
WASO (%)	13.28 (16.84)	5.28	18.76 (19.37)	12.66	8.89 (12.56)	4.13	**0.066**#	0.296	**0.062**
NREM 1 (%)	32.53 (20.25)	32.05	39.91 (30.66)	39.64	32.77 (21.40)	30.18	0.448		
NREM 2 (%)	47.43 (21.92)	49.04	44.75 (28.97)	44.97	40.74 (19.30)	44.24	0.608		
NREM 3 (%)	17.52 (17.47)	14.84	9.49 (19.51)	0.00	20.68 (24.51)	14.82	0.143#		
REM (%)	2.53 (8.17)	0.00	5.85 (15.96)	0.00	5.81 (16.18)	0.00	0.495#		
**Sleep architecture of entire nap excluding so-tDCS/sham intervals**									
Total Sleep Time (min)	43.30 (12.10)	40.75	39.77 (14.17)	38.50	45.07 (16.01)	50.25	0.345		
Sleep Period Time (min)	59.11 (13.43)	63.00	55.57 (12.62)	54.50	54.77 (16.90)	59.00	0.727		
Sleep Onset latency (min)	9.30 (6.61)	6.75	12.82 (9.31)	10.25	9.34 (10.36)	5.25	0.147#		
WASO (%)	25.74 (17.15)	31.20	28.29 (18.53)	29.35	17.04 (14.61)	13.16	0.170#		
NREM 1 (%)	36.47 (15.21)	37.01	8.36 (23.60)	51.35	40.70 (20.75)	41.79	**0.074**	0.459	0.382
NREM 2 (%)	52.59 (18.10)	60.53	43.69 (20.31)	47.10	42.28 (16.94)	42.37	0.108#		
NREM 3 (%)	9.40 (11.80)	5.00	4.99 (9.78)	0.00	13.90 (17.03)	9.42	**0.036**#*	0.601	**0.046***
REM (%)	1.55 (5.11)	0.00	2.97 (8.22)	0.00	3.13 (10.82)	0.00	0.396#		
Sleep efficiency (%)	60.50 (18.55)	53.47	56.56 (20.53)	54.20	62.55 (21.77)	66.77	0.514		
Sleep latency N1 (min)	9.71 (7.49)	6.75	12.82 (9.31)	10.25	9.66 (10.41)	5.25	0.147#		
Sleep latency N2 (min)	14.36 (9.23)	14.50	20.39 (14.14)	16.75	16.46 (12.26)	13.75	**0.049**#*	0.694	0.848
Sleep latency N3 (min)	24.88 (8.23)	30.50	35.75 (19.15)	38.25	37.88 (19.32)	29.25	0.478		

Sleep stages presented as percentage of total sleep time. MD = median, SD = standard deviation. Sleep efficiency = total sleep time divided by total duration of the hypnogram. WASO = wake after sleep onset, WASO (%) = WASO (min) divided by sleep period time. Differences in sleep architecture were statistically compared between conditions with repeated-measures ANOVA, followed by planed contrasts (cathodal vs. sham; anodal vs. sham) in case of significant difference.

Significant effects (*p < 0.05) and trends towards significance are marked bold.

Please note: Stimulation/sham time periods are not included in the total sleep time, sleep period time, and sleep onset latency.

Friedman test followed by Wilcoxon signed-rank test due to skewed distribution.
