# Supplementary material for: Polarity-dependent modulation of sleep oscillations and cortical excitability in aging
Source: Front Aging Neurosci. 2026 Jan 15;17:1704130. doi: 10.3389/fnagi.2025.1704130 (PMC12852367; doi:10.3389/fnagi.2025.1704130)
Supplement: Supplementary file 6 [file Table_6.pdf]

Table S6. Number of stimulation/sham blocks

Frequency of stimulation block counts during the nap (n)	cathodal so-tDCS	anodal so-tDCS	sham
7 blocks	2	3	3
8 blocks	4	0	1
9 blocks	2	1	2
10 blocks	1	4	1
11 blocks	3	4	2
12 blocks	1	3	1
13 blocks	3	0	4
14 blocks	2	0	0
15 blocks	4	7	8

The frequency of stimulation/sham blocks was determined by the participant ‘s sleep in each condition.
